# Harvesting metadata in clinical care: a crosswalk between FHIR, OMOP, CDISC and openEHR metadata

**DOI:** 10.1038/s41597-022-01792-7

**Published:** 2022-10-28

**Authors:** Caroline Bönisch, Dorothea Kesztyüs, Tibor Kesztyüs

**Affiliations:** Medical Data Integration Center, Department of Medical Informatics, University Medical Center Göttingen, Robert-Koch-Str. 40, 37075 Göttingen, Germany

**Keywords:** Health care, Public health

## Abstract

Metadata describe information about data source, type of creation, structure, status and semantics and are prerequisite for preservation and reuse of medical data. To overcome the hurdle of disparate data sources and repositories with heterogeneous data formats a metadata crosswalk was initiated, based on existing standards. FAIR Principles were included, as well as data format specifications. The metadata crosswalk is the foundation of data provision between a Medical Data Integration Center (MeDIC) and researchers, providing a selection of metadata information for research design and requests. Based on the crosswalk, metadata items were prioritized and categorized to demonstrate that not one single predefined standard meets all requirements of a MeDIC and only a maximum data set of metadata is suitable for use. The development of a convergence format including the maximum data set is the anticipated solution for an automated transformation of metadata in a MeDIC.

## Introduction

Since humans began sorting and categorizing information and objects, metadata provided important alignment of information objects. Metadata are defined as data about data^1^. They describe information objects with regard to source, type of creation, structure, status, level and semantics. An information object can be either a data including a coded value or instance identifier, or a list of several dates, or an entire database with various dependencies^1^. Using metadata, related data can be reused, organized, described, validated, searched and queried. In the medical field, the provision, reuse and preservation of information is essential to ensure the best possible treatment of a patient as well as answering research questions. As Hegselmann *et al*. stated “Individuals with very specific characteristics could be identified, which is mandatory for personalized medicine as well as epidemiological and clinical studies, but also general big data applications would be possible”^2^. Retrospective acquired data, especially if largely available, provides opportunities not only to predict but detect e.g. novel risks and therapeutic options on an individual level (precision medicine)^3^. Consequently, the subordinate metadata are predominant to prepare the basis for combining and transforming the data by providing metainformation to enable linkage of information from different data sources. This goes to show in which way metadata benefits the medical sector.

The FAIR Principles, postulated in 2016^4^, suggest that the reuse of (meta)data is of great importance in the context of medical research. The management of data with the corresponding application of metadata provides multiple opportunities for high-quality data analyses and subsequent high impact publications. Metadata for research data is also gaining importance in light of the increasing requirement of journals to make primary data from published research publicly available^5^. The FAIR Principles are divided into the categories Findable, Accessible, Interoperable and Re-Usable. Metadata are explicitly named in all four categories. Therefore metadata act as important building blocks for making information accessible and usable.

However, Dugas *et al*. acknowledge that most forms and item catalogs from healthcare research studies in Germany do not comply to these FAIR Principles and cannot be easily found and are therefore not re-usable^6^. This is due to the fact that forms are sometimes not allowed to be published, because of permission restrictions or they are not published based on interoperability points of view, e.g. without an identification number or accompanying metadata, and remain in a paper tomb. As stated in the article, it is important to publish metadata with the data, as this characterizes a first step towards open data^6^.

Mainly in the biomedical field, it is noticeable that there were and still are implementations where the importance of metadata is highlighted^7^. Different consortia and working groups provide their approaches to utilize metadata with regards to re-usability, accessibility and findability^8,9^. Both aforementioned articles adopt the paradigm that qualitative metadata is helpful to retrieve, acquire and utilize metadata. The working group referenced in^8^ proposes the potential of ontology concepts to annotate metadata making them easier to be found and semantic specific, resulting in a strong descriptor of the resource contents.

Gonçalves *et al*.^9^ developed a software that pulls information from metadata records and analyses the information whether it is complete and correct according to given specifications (right format and legitimate content).

Data integration from heterogeneous source data systems is a major challenge, not only in the biomedical field. Initially, each system has very different metadata attributes that must be taken into account. The structures of the data used are specifically designed for the respective source data system. This can make it difficult to reuse data, which was collected within specific source systems, due to proprietary reasons.

Canham and Ohmann describe that metadata can be divided into two parts. On the one hand, there is intrinsic metadata, which is permanent and unchangeable^10^. For instance, metadata such as the date/timestamp and the performing clinician, as well as the status (active, postponed, complete) of a clinical examination, is considered intrinsic metadata.

On the other hand, they identify provenance metadata, which represents localization or history, like the data lifecycle state (creation, processing, analysis, preservation, access, reuse), the data custodian or the method of data collection. Provenance metadata is subject to change, because of its nature to provide information about non-static knowledge. Both intrinsic and provenance metadata are required for searching and uniquely identifying data. The variability of data in routine clinical practice makes the use of a unified metadata schema complicated, but nonetheless Canham and Ohmann proposed a common metadata scheme within the “protocol-driven clinical research”, that would be applicable to any information system. As they note, it is more beneficial if the data and corresponding metadata remain in their original relational form and are converted to the desired target format FHIR, openEHR or OMOP using a parser^10^. An appropriate crosswalk between the individual metadata elements of the respective standards is of utmost importance to obtain the most fine-grained result possible with a maximum set of metadata elements.

Metadata harvesting describes a process to combine metadata from different data storages, archives or repositories and store them in a central database schema. The data that is harvested in this work is derived from the Medical Data Integration Center (MeDIC) of the University Medical Center Göttingen (UMG). The University Medical Center is a hospital of maximum care and extensive sources of medical data. The MeDIC joins medical information and their corresponding metadata from hospital information systems and clinical research data bases (which include, inter alia, data from studies and registries, such as case report forms, patient reported outcomes or findings) in a data warehouse. It involves data from datasets with different (meta)data types and longitudinal data collection, as well as data integration. The data and corresponding metadata are stored in a relational database, which underlies the data warehouse of the MeDIC. The metadata is kept in a distinct table separated from the data, connected via a primary/foreign key to the tables of data. Therefore, it is possible to store metadata in a n-dimensional repository in the same format The medical source data from the hospital and department information systems are pseudonymized and transformed into the internal harmonized data format of the MeDIC. During the process of harvesting metadata within the Extract-Transform-Load process, metadata is extracted and loaded via a MeDIC-specific data protocol, preventing duplicates. The data warehouse of the MeDIC anticipates to connect all available data sources of the UMG as part of an ongoing process.

In this article, we aim to provide a crosswalk between the formats mentioned above and try to convey them as accurate as possible.

## Results

For the purpose of this research project, the specifications for the data formats CDISC, OMOP, openEHR and FHIR are examined. For every data format all corresponding metadata items are extracted and contrasted.

Following the conception of the metadata crosswalk, the next phase includes the identification of metadata items with high relevance for the MeDIC.

Taking into account the results from the literature research, e.g. the FAIR Principles and requirements (which are immanent to the MeDIC structure), essential metadata items are decided upon.

Within the FAIR Principles, the principles F1, F3, F4, A2, I3 and R1.1 have been taken into account, as they directly relate to metadata^4^. F1 postulates that (meta)data has to be assigned with a globally unique and persistent identifier. F3 involves metadata that includes identifier, which clearly and explicitly describes the corresponding data, whereas F4 claims metadata to be indexed in a searchable resource. According to A2 the metadata has to be accessible, even when the data are not obtainable. I3 contains that metadata must include a qualified reference to other metadata. Finally, R1.1 suggests that metadata has to be released with a clear and accessible data usage license^4^. The requirements for the MeDIC resulted from a requirements analysis, that was conducted at the beginning of the development of the MeDIC in 2019.

Table 4 shows the resulting matrix of mappings including the prioritization of the metadata items.

**Fig. 1:**
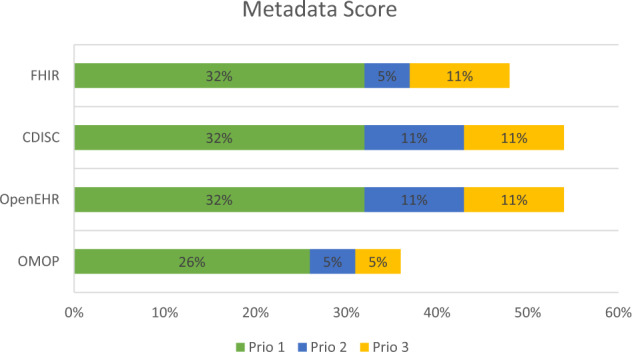
Qualitative priority scoring of metadata required by the MeDIC and quantitative coverage in the different data formats FHIR, CDISC, OpenEHR and OMOP.

In Fig. 1, the scores regarding prioritization are calculated for the individual data formats and compared graphically in the following grouped bar chart.

As can be seen from the previous illustrations, none of the data formats fulfill all required MeDIC-inherent criteria.

As stated, an entire transformation is not possible because of the different premises the individual formats are based on. CDISC, for example, has added structures, that build upon the ODM format and form in conjunction the basis for study documentation in clinical care. OpenEHR on the other side is designed for the storage of medical data in an EHR, while FHIR is intended for the exchange of data between different institutions. Whereas OMOP provides a common data format to unify data from different databases. It is shown, that none of the data formats include all metadata, which is required to successfully operate the MeDIC for the purpose of reliable data management. So we propose a specific convergence format, which bypasses the described challenges.

**Fig. 2:**
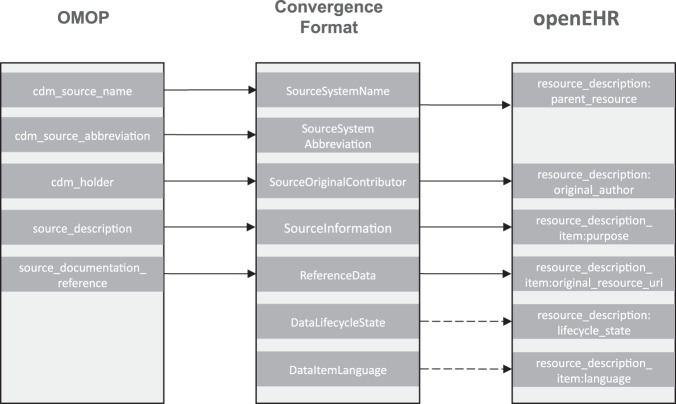
Example of an excerpt of metadata items from OMOP and openEHR, showing how the convergence format can avoid loss of information during the metadata transformation. Dotted arrows show data flow with NULL-values.

Figure 2 shows an example by providing an excerpt of two data formats OMOP and openEHR. It illustrates how the convergence format can incorporate metadata items of different formats and avoid loss of information by providing metadata items of the target format even if it is not part of the source data format. For example the metadata item *cdm_source_abbreviation* has no exact match in openEHR metadata. Without the convergence format, the information would have been lost, because it would be no longer represented in the openEHR metadata items after the transformation.

Additionally openEHR accepts metadata items such as resource_description:lifecycle_state and resource_description_item:language, which have no match within the OMOP metadata. Naturally these two metadata items would have not been created within the transformation process, because OMOP doesn’t provide the equivalent structure. The convergence format is therefore the best solution to provide and maintain the format structure, by creating the items within the transformation process and filling them with NULL-values, if the source format doesn’t provide any input values.

## Discussion

The literature search revealed that the topic of metadata is of high importance in medical and biomedical informatics. A fundamental problem, however, is the definition of metadata. Ulrich *et al*. examines the literature on the definition and classification of metadata and points out the fact, that there is no clear explanation of the term “metadata”. Furthermore, the article shows how the definition of matching, mapping and transformation of metadata also differs in the literature. Overall, the article points out possible problems that can result from the heterogeneous understanding of the term^11^.

Some authors previously showed the possibility of transforming single data formats into each other. However, the transformation between more than two data formats within a metadata crosswalk has not yet been performed. The works of Doods, Neuhaus & Dugas^12^, and Bruland & Dugas^13^ for example, show the possibilities of a transformation of openEHR or FHIR into CDISC ODM. Within the MeDIC, however, structured as well as unstructured data and metadata are to be considered, which contradicts a mere use of CDISC ODM as target format.

The use of different data formats and the associated metadata formats in health care results in heterogeneity of the metadata items. This leads to the fact that occasionally a complete match between the data fields is unachievable, resulting in inequivalence. Be it that the data formats support different application areas or that they allow different degrees of freedom in the development of extensions.

The findings presented in this article show that metadata items from different standard formats meet the requirements to be transformed into one another with few adaptations, because of the previously mentioned challenges of a metadata crosswalk.

In the next stage, the convergence format will be further developed and an automated crosswalk between the different data formats to and from the convergence format will be established. This convergence format comprises both MeDIC inherent metadata items and all items from the crosswalk of the four data formats depicted in Table 2.

This maximum set of metadata items will be the requisite to fulfill the gap between the metadata currently captured in hospital information systems and the derivatives of data needed in research, to be able to provide metadata to researchers in any data format. Additionally, the quality of the harvested metadata has to be evaluated. If providing metadata to researchers, they must also be assured that it is of high quality and allows safe evaluations. Therefore, a quality assessment schema will be developed. This assessment should lead to a visualization of the metadata quality, which is then made available to the researchers. This visualization enables a researcher to easily recognize and evaluate the data quality and whether the data is suitable for this research purpose.

## Methods

### Literature research

To assess the field of existing metadata standards a literature research was conducted using PubMed and Embase via Ovid.

Table 1. shows the search steps and partial results of the literature search exemplarily in PubMed. The results of this search were combined with a second search query using the same search strategy within Embase.

**Table 1:** Search strategy in PubMed on 18.05.2022.

No.	Search Step	Results
#1	“metadata”[MeSH Terms]	413
#2	“standard*“[Title/Abstract] OR “open standard*“[Title/Abstract]	1,415,975
#3	“data warehousing”[MeSH Terms] OR “health information interoperability/standards”[MeSH Terms] OR “health information exchange/standards”[MeSH Terms] OR information storage and retrieval/methods”[MeSH Terms]	17.042
#4	“metadata repository”[Title/Abstract] OR “data integration”[Title/Abstract]	4.071
#5	“Medical Records Systems, Computerized”[MeSH Terms]	45.478
#6	“open EHR”[Title/Abstract] OR “CDISC”[Title/Abstract] OR “FHIR”[Title/Abstract] OR “OMOP”[Title/Abstract]	587
#7	#1 AND (#2 OR #3 OR #4 OR #5 OR #6	205

For the purpose of adequately selecting and evaluating the results of the literature search, articles proposing necessary metadata for data exchange and processing (search criterion one), as well as (meta)data format specification (search criterion two) and articles describing already existing metadata crosswalks (search criterion three) or approaches of transforming/mapping metadata formats into one another (search criterion four), where taken into consideration.

The search in both bibliographic databases yielded 517 results, of which 71 duplicates were removed automatically in Refworks, and 446 references remained. The relevance of every result was examined by scanning the associated title and abstract. After this examination, 60 articles with high importance were left, and full text of these was obtained. After reading the full text, 12 articles were deemed not to be suitable for the purpose of this research. The reference lists of all included articles were scanned for further important publications. Finally, the remaining articles were studied completely and evaluated in relation to the search criteria used to select, evaluate and prioritize the results of the literature search. The proposed FAIR Principles^4^ and the documentation of the included data formats where the core answers to the first search criterion, while the documentation also answered the second search criterion and served as the preface for the development of the crosswalk.

Kock-Schoppenhauer *et al*. and Bruland and Dugas showed first elaborations of one-to-one transformations between different data formats, related to search criterion three^13,14^. while^15^ and^16^ supplied a founded overview over the procedure of a crosswalk.

### Metadata crosswalk

In order to make the medical data collected in the data integration center available to researchers, the metadata are supposed to be made accessible in data formats which are frequently used. Currently the data formats to be supported in the MeDIC include OMOP, openEHR, FHIR and CDISC. This allows researchers to be offered a choice of target data formats. To enable this selection a metadata crosswalk is built.

A metadata crosswalk entails a chart or map which depicts elements from different standards or formats and groups equivalent elements^15^. Crosswalks allow to transform elements from on format to another^16^.

The excerpt of the developed metadata crosswalk for these four formats is depicted in Table 2. The complete crosswalk can be found in the Supplementary Table 1 within the Supplementary material of this manuscript.

**Table 2:** Comparison of metadata from different data formats frequently used in healthcare information systems and medical research.

Meaning of local metadata items	OMOP	openEHR	FHIR	CDISC
Version of the metadata	metadata_concept_id	versionID	Meta.versionID	ODM/Study/MetaDataVersion
Identifier of the type of information	metadata_type_concept_id			
Name of the metadata version	name			ODM/Study/MetaDataVersion/Name
Metadata value as string	value_as_string			
Metadata value as concept	value_as_concept_id			
Date of the metadata creation	metadata_date		DataRequirement	ODM/AsOfDatetime
Datetime of the metadata creation	metadata_datetime		Meta.lastUpdated	ODM/AsOfDatetime
Full name of the source	cdm_source_name	resource_description:parent_resource	Meta.profile	def:Origin
Abbreviation of the source name, if applicable	cdm_source_abbreviation			def:Origin
contributor or publisher of the source data	cdm_holder	resource_description:original_author/resource_description:original_publisher	Contributor	def:Origin

Note. OMOP Observational Medical Outcome Partnership, openEHR open Electronical Health Records, FHIR Fast Healthcare Interoperability Resources, CDISC Clinical Data Interchange Standards Consortium.

During a transformation, data fields from the input format sometimes have to be split or merged in order to retain the semantic meaning of the metadata in the target format.

As a result of the above challenges, a loss of information can occur. In order to avoid this, a convergence format, has to be used that includes and stores a maximum set of metadata fields. To establish the convergence format a defined metadata crosswalk serves as the objective of this work.

### Prioritization

After the crosswalk has been executed, metadata items which are specifically important to the MeDIC are identified. These metadata are determined based on the literature review and data format specifications. Then, the items meeting the inherent requirements of the MeDIC are categorized. For this purpose, a split into three categories is chosen. Category 1 includes items that are of immense importance for the operation of the MeDIC and for the provision of data. Category 2 includes objects that span a medium importance but are requisite for data privacy and consent. Category 3 consists of metadata with the lowest priority, which are key for additional context information and language specification. An urgency of the corresponding items is not to be considered. For this reason, the priority dimension only includes the importance of the items for the MeDIC.

After prioritization, the scores for the individual data formats are calculated to show which data formats cover items of priority categories 1 and 2 as extensively as possible.

Table 3 shows the identified metadata items and the associated prioritization.

**Table 3:** Essential metadata required in the MeDIC and respective priority level.

Metadata Item	Priority	Description
MetadataID	1	Unique and persistent identifier of the metadata
MetadataDate	1	Date of the metadata creation
AffiliateDatasetID	1	Globally unique identifier of the data, which the metadata is associated with
MetadataVersion	1	Version of the Metadata
ReferenceData	1	References to other data via name or description
ReferenceMetadata	1	References to other metadata via name or description
DataLifecycleState	2	State of the data during its lifecycle (creation, processing, analysis, preservation, access, reuse)
UsageLicense/Copyright	1	clear and accessible data usage license
UsageContext	3	Context in which the data should be used
SourceSystemName	1	Explicit name of the Source System
SourceSystemVersion	1	Version of the Source System when recording the (meta)data
SourceInformation	3	Additional information about the source of the data
SourceOriginal Contributor	2	Contributor of the Source data
VestingPeriod	3	Availability of data to other researcher outside the study during the time of the study
ConsentType	2	Type of patient consent (i.e., broad consent, study specific consent)
ConsentValidation	1	Validity period of the consent
ConsentVerification	1	Physical signature of the patient and start of the validity period
ConsentModule	2	Exact parts of the consent, to which the patient consented to
DataItemLanguage	3	Language of the data items

Note Priority level 1 = high, 2 = medium, 3 = low.

**Table 4:** Matrix of mapping of priorities to metadata items.

Metadata Item (Priority level)	OMOP	openEHR	FHIR	CDISC
MetadataID (1)	metadata_ concept_id	versionID	Meta.versionID	ODM/Study/Metadata Version
MetadataDate (1)	metadata_date		DataRequirement	ODM/AsOfDatetime
AffiliateDatasetID (1)	source_ description_ reference	resource_ description_item:original_resource	DataRequirement. Profile	def:Origin
MetadataVersion (1)	metadata_ concept_id	versionID	Meta.versionID	ODM/Study/Metadata Version
ReferenceData (1)		resource_ description: references	RelatedArtifact	def:LeafElement
ReferenceMetadata (1)				
DatalifecycleState (2)	resource_ description: lifecylce_state	def:AnnotatedCRF		
UsageLicense/Copyright (1)	resource_ description: copyright			
UsageContext (3)	resource_ description_item:use	UsageContext	StudyDescription	
SourceSystem Name (1)	cdm_source_name	resource_ description: parent_resource	Meta.profile	def:Origin
SourceSystem Version (1)				
SourceInformation (3)	source_description	resource_ description_item:purpose	DataRequirement.type	def:Origin
SourceOriginalContributor (2)	cdm_holder	resource_ description: original_author & resource_ description: original_publisher	Contributor	def:Origin
VestingPeriod (3)				
ConsentType (2)				
ConsentValidation (1)				
ConsentVerifikation (1)				
ConsentModule (2)				
DataItemLanguage (3)	resource_ description_item:language			

Note. OMOP Observational Medical Outcome Partnership, openEHR open Electronical Health Records, FHIR Fast Healthcare Interoperability Resources, CDISC Clinical Data Interchange Standards Consortium.

The prioritization is divided into categories 1, 2 and 3. Category 1 contains items with the highest priority, while category 3 contains the items with the lowest priority. 19 Metadata items are identified, which will contribute to the sustainability of the MeDIC in terms of data usage and exchange. Metadata items resulting from the FAIR Principles are assigned the highest priority because of its high importance to make data findable, accessible, interoperable and re-usable.

The mapping of priorities to metadata items is then applied to the metadata of the four different data formats.

## Supplementary information

Supplementary Table 1 Metadata Crosswalk

## Data Availability

All data generated or analyzed during this study are included in this article and is referenced in^17^.

## Code availability

During this study no custom code was used.
