# Supplementary material for: Harvesting metadata in clinical care: a crosswalk between FHIR, OMOP, CDISC and openEHR metadata
Source: Sci Data. 2022 Oct 28;9:659. doi: 10.1038/s41597-022-01792-7 (PMC9616884; doi:10.1038/s41597-022-01792-7)
Supplement: Supplementary file 1 — Supplementary Table 1 Metadata Crosswalk [file 41597_2022_1792_MOESM1_ESM.docx]

**Supplementary Information**

Table of Contents

[Supplementary Table 1 Metadata Crosswalk 2](#_Toc116641517)

| Supplementary Table 1 Metadata Crosswalk | | | | |
| --- | --- | --- | --- | --- |
| Meaning of local metadata items | OMOP | openEHR | FHIR | CDISC |
| Version of the metadata | metadata_concept_id | versionID | Meta.versionID | ODM/Study/MetaDataVersion |
| Identifier of the type of information | metadata_type_concept_id |  |  |  |
| Name of the metadata version | name |  |  | ODM/Study/MetaDataVersion/ Name |
| Metadata value as string | value_as_string |  |  |  |
| Metadata value as concept | value_as_concept_id |  |  |  |
| Date of the metadata creation | metadata_date |  | DataRequirement(DataRequirement.dateFilter, DataRequirement.dateFilter.path, DataRequirement.dateFilter.searchParam, DataRequirement.dateFilter.value) | ODM/AsOfDatetime |
| Datetime of the metadata creation | metadata_datetime |  | Meta.lastUpdated | ODM/AsOfDatetime |
| Full name of the source | cdm_source_name | resource_description:parent_resource | Meta.profile | def:Origin |
| Abbreviation of the source name, if applicable | cdm_source_abbreviation |  |  | def:Origin |
| contributor or publisher of the source data | cdm_holder | resource_description: original_author/Resource_description:original_publisher | Contributor (Contributor.type, Contributor.name) | def:Origin |
| Description of the source data origin and purpose for collection | source_description | resource_description_item:purpose | DataRequirement.type | def:Origin |
| External reference to the location of the source | source_documentation_ reference | resource_description_item:original_resource_uri | DataRequirement.profile | def:Origin |
| External reference to ETL specification documentation and ETL source code | cdm_etl_reference |  |  | ProtocolName |
| Date for which the source data are most current (e.g. last day of data capture) | source_release_date | timestamp of committal or creation of the item |  |  |
| Date when the common data model was instantiated | cdm_release_date |  |  |  |
| Version of the common data model | cdm_version |  |  |  |
| Version of the vocabulary used | vocabulary_version |  |  | CodeList, CodeListItem |
| Information about the data lifecycle state |  | resource_description: lifecycle_state |  | def:AnnotatedCRF |
| Namespace of originals author’s organisation |  | resource_description:original_ namespace |  |  |
| Other contributors to the source |  | resource_description:other_ contributors |  |  |
| Namespace of current custodian organisation |  | resource_description: custodian_namespace |  |  |
| Text name of current custodian organisation |  | resource_description: custodian_organisation | Contributor.Contact |  |
| Copyright statement |  | resource_description: copyright |  |  |
| Licence information |  | resource_description:license |  |  |
| List of acknowledgements of other terminology codes, ontology ids referenced |  | resource_description:ip_acknowledgements |  |  |
| Reference list of material to the artifact |  | resource_description:references | RelatedArtifact ( RelatedArtifact.type; RelatedArtifact.label; RelatedArtifact.display; RelatedArtifact.citation; RelatedArtifact.url; RelatedArtifact.document; RelatedArtifact.resource) | def:LeafElement |
| URI to which this resource belongs |  | resource_description:resource_ package_uri |  |  |
| Details of the conversion process/generation of the model |  | resource_description:conversion_ details |  |  |
| Additonal resource metadata |  | resource_description:other_details | DataRequirement.mustSupport | def:CommentDef |
| Details of all parts of resource description in natural language |  | resource_description:details | DataRequirement.subject | StudyDescription |
| Localised language of the data items |  | resource_description_item:language |  |  |
| Keywords describing the resource |  | resource_description_item:keywords |  |  |
| Context or use of the resource |  | resource_description_item:use | UsageContext (UsageContext.code; UsageContext.value) | StudyDescription |
| Misuse of the resource |  | resource_description_item:misuse |  |  |
| Additional metadata of the data item |  | resource_description_item: other_details | DataRequirement.codeFilter.searchParam; DataRequirement.codeFilter.valueSet; DataRequirement.codeFilter; DataRequirement.codeFilter.path; DataRequirement.codeFilter.code | def:ValueListDef |
|  |  |  | DataRequirement.limit |  |
| Sorting specifications of required data (ascending, descending) |  |  | DataRequirement.sort, DataRequirement.sort.path; DataRequirement.sort.direction |  |
| Definition of Events when to evaluate a data item |  |  | TriggerDefinition ( TriggerDefinition.type, TriggerDefinition.name; TriggerDefinition.timing[x]; TriggerDefinition.data; TriggerDefinition.condition) |  |
| Expression, to generate a value |  |  | Expression (Expression.description; Expression.name; Expression.language; Expression.expression; Expression.reference) |  |
| Supplemental Documentation |  |  |  | def:SupplementalDoc |
| Definition of standard used for the data item |  |  |  | Def:Standards(specifies name, type, status) |
| Definition of the obtainability of data from a data collection |  |  |  | MethodDef |
| Comparison of metadata from different data formats frequently used in healthcare information systems and medical research ***Note****.* OMOP Observational Medical Outcome Partnership, openEHR open Electronical Health Records, FHIR Fast Healthcare Interoperability Resources, CDISC Clinical Data Interchange Standards Consortium | | | | |
